# ACUTE ABDOMEN IN INTENSIVE CARE UNIT: ETIOLOGY, COMORBIDITY AND SEVERITY OF 1,523 PATIENTS

**DOI:** 10.1590/0102-672020230060e1778

**Published:** 2023-12-08

**Authors:** Geraldo Fernandes de ALMEIDA FILHO, Pedro Paulo Costa e SILVA, Murilo Tavares VALVERDE FILHO, Maria Clara Alves MORAIS, Paulo Bravo de Oliveira CHAGAS, Ricardo Azevedo Cruz D’OLIVEIRA, Liana CODES, Paulo Lisboa BITTENCOURT

**Affiliations:** 1 Escola Bahiana de Medicina e Saúde Pública, Medical School – Salvador (BA), Brazil;; 2 Hospital Português, Gastroenterology and Hepatology Unit – Salvador (BA), Brazil

**Keywords:** Abdomen, acute, Intensive care units, Critical care, APACHE, Comorbidity, Mortality, Abdome agudo, Unidades de terapia intensiva, Cuidados críticos, APACHE, Comorbidade, Mortalidade

## Abstract

**BACKGROUND:**

Clinical features and outcomes of patients admitted to the intensive care unit due to acute abdomen are important to be investigated.

**AIMS:**

To evaluate the outcomes of critically ill subjects with acute abdomen according to etiology, comorbidity and severity.

**METHODS:**

Outcomes of 1,523 patients (878 women, mean age 66±18 years) consecutively admitted to a specialized gastrointestinal intensive care unit with different causes of acute abdomen from January 2012 to December 2019, were retrospectively evaluated according to etiology, comorbidity and severity.

**RESULTS:**

The most common causes of acute abdomen were obstructive and inflammatory, particularly large bowel obstruction (27%), small bowel obstruction (18%) and acute pancreatitis (17%). Overall mortality was 13%. Surgery was required in 34% of patients. Median length of stay in the hospital was 9 [1-101] days. On univariate analysis mortality was significantly associated with age, APACHE II, Charlson comorbidity index, requirement for surgery and malignancy (p<0.0001), but only APACHE II, Charlson comorbidity index and surgical interventional remained significant on multivariate analysis.

**CONCLUSIONS:**

Critically ill patients admitted to the intensive care unit with acute abdomen constitute a heterogeneous group of subjects with different prognosis. Mortality is more related to the severity of the disease, comorbidity and need for surgery than to the etiology of the acute abdomen.

## RESUMO

**RACIONAL:**

As características clínicas e os desfechos dos pacientes internados na unidade de terapia intensiva devido ao abdômen agudo são importantes serem investigados.

**OBJETIVOS:**

Avaliar os desfechos de indivíduos gravemente doentes com abdômen agudo de acordo com etiologia, gravidade e comorbidade.

**MÉTODOS:**

Os desfechos de 1.523 pacientes (878 mulheres, média de idade 66±18 anos) que foram previamente admitidos em uma unidade de terapia intensiva especializada em doenças gastrointestinais, com diferentes causas de abdômen agudo entre janeiro de 2012 e dezembro de 2019, foram avaliados retrospectivamente segundo etiologia, comorbidade e gravidade.

**RESULTADOS:**

As causas mais comuns de abdômen agudo foram obstrutivas e inflamatórias, com destaque para obstrução em colon (27%), em intestino delgado (18%) e pancreatite aguda (17%). A mortalidade geral foi de 13%. A cirurgia foi necessária em 34%. A média de permanência no hospital foi de 9 [1-101] dias. Na análise univariada a mortalidade foi significativamente associada à idade, APACHE II, índice de comorbidade de Charlson, necessidade de abordagem cirúrgica e presença de malignidade (p<0,0001), mas apenas APACHE II, índice de comorbidade de Charlson e intervenção cirúrgica permaneceram significativos na análise multivariada.

**CONCLUSÕES:**

Pacientes internados na unidade de terapia intensiva com abdômen agudo constituem um grupo heterogêneo de indivíduos com prognóstico diferente. A mortalidade está mais relacionada com a gravidade da doença, comorbidade e necessidade de cirurgia do que com a etiologia do abdome agudo.

## INTRODUCTION

Acute abdominal pain (AAP) or acute abdomen (AA) accounts for 4–9.1% of all visits to the emergency department (ED)^5,6,24^. Clinical investigation of patients with AA are crucial for guiding further evaluation with laboratory tests and imaging^12,19,20^, in as much as benign non-specific abdominal pain (NSAP) still remains the most common subjacent diagnosis of AA^5-7,11,14^. Other common causes of non-traumatic AA include nephrolithiasis, cholelithiasis and/or cholecystitis (CC), acute appendicitis, acute pancreatitis (AP), acute diverticulitis, small (SBO) and large bowel obstruction (LBO), perforated hollow viscus and mesenteric ischemia^1,5-7,10,15,17,26,31^.

Approximately 2/3 of those patients are discharged from the ED, particularly those with nephrolithiasis, NSAP and non-complicated inflammatory acute abdomen, but patients with life-threatening disorders with actual or impending organ failure or significant comorbidity due to a higher risk of complications and death are usually admitted to intermediate or intensive care units (ICU) for real time monitoring of organ function, management of associated sepsis or hemodynamic optimization before or after urgent or even elective surgery^12,19^.

One recent Brazilian study has evaluated the incidence and mortality of patients with AA admitted to the hospital through the Brazilian Unified Public Health System (SUS)^18,22^. The most common causes were CC, acute appendicitis, AP, complications of gastric and duodenal ulcers, acute diverticulitis and inflammatory bowel disease (B),(C). The authors have noted an increase in the frequency of acute appendicitis, AP and acute diverticulitis over the years. Mortality due to complications of gastroduodenal ulcers, acute diverticulitis and AP was higher when compared to other causes of AA^18^.

There are few studies concerning the most frequent causes of AA in patients admitted to the ICU and their outcomes in respect to requirement of surgical intervention, length of stay (LOS) and mortality^13^.

The purpose of this study was to evaluate the epidemiology and outcomes of patients with AA admitted to the ICU in a tertiary care hospital in Brazil as well as to investigate risk factors associated with mortality.

## METHODS

All patients admitted to the Gastroenterology and Hepatology Unit of the Hospital Português in Salvador (BA), with the diagnosis of AA from January 2012 to December 2019, were retrospectively analysed. This facility is an intensive gastrointestinal ICU specialized in management of critically ill patients with gastrointestinal disorders, such as AA, gastrointestinal hemorrhage, decompensated cirrhosis and acute liver failure, as well as patients in the postoperative period of major abdominal surgery.

The diagnosis of AA was suspected, by the attending physician, based on clinical, laboratory, imaging data and surgical findings whenever surgery was required. It was further categorized as inflammatory (IAA), obstructive (OAA), perforated (PAA), traumatic (TAA) or vascular (VAA) AA, as previously described^8,16^.

All patients were followed according to the hospital protocol^2^, which has been constantly updated according to international guidelines.

Patients in palliative care were excluded from the analysis. Patients were followed until death or hospital discharge. The primary endpoint was in-hospital mortality.

The study was approved by the Research Ethics Committee of Hospital Português (number 26195719.0.0000.5029).

### Statistical analysis

The variables are presented in text and tables as numbers and percentage. Clinical and laboratory features were compared using the chi-square or Fisher’s test. Continuous variables were reported as mean and standard deviation (SD) or as median and interquartile range, respectively, whether the distribution was normal or skewed, using the Student *t* test or the Mann-Whitney U test. Variables associated with mortality at univariate analysis with a p-value of <0.10 were entered in multivariate logistic regression modeling using stepwise elimination. The software used for analysis was the Statistical Package for Social Sciences (SPSS Inc., Chicago, IL, EUA), version 14.0 for Windows.

## RESULTS

One thousand five hundred and twenty-three patients (878 women, mean age 66±18 years) were admitted to the ICU with an AA between January 2012 and December 2019. The clinical and laboratory data and outcomes of those subjects are in Table 1. 

**Table 1:** Clinical and laboratory features and outcomes of patients admitted to the intensive care unit with acute abdomen (n=1,523)

Demographics	
Age (years)	66±18
Gender (n%)	
Male	645 (42)
Female	878 (58)
Clinical and laboratory features	
APACHE II score	11±6
Charlson comorbidity index	4 [0–13]
Concurrent malignancy	349 (23%)
Classification of acute abdômen (n%)	
Obstructive	709 (47)
Inflammatory	692 (45)
Vascular	51 (4)
Perforated	39 (3)
Traumatic	16 (1)
Hemorrhagic	6 (1)
Outcomes	
Surgery	517 (34%)
Length of stay	9 [1–101]
Mortality	196 (13%)

APACHE II: acute physiology and chronic health evaluation II.

**Figure 1:**
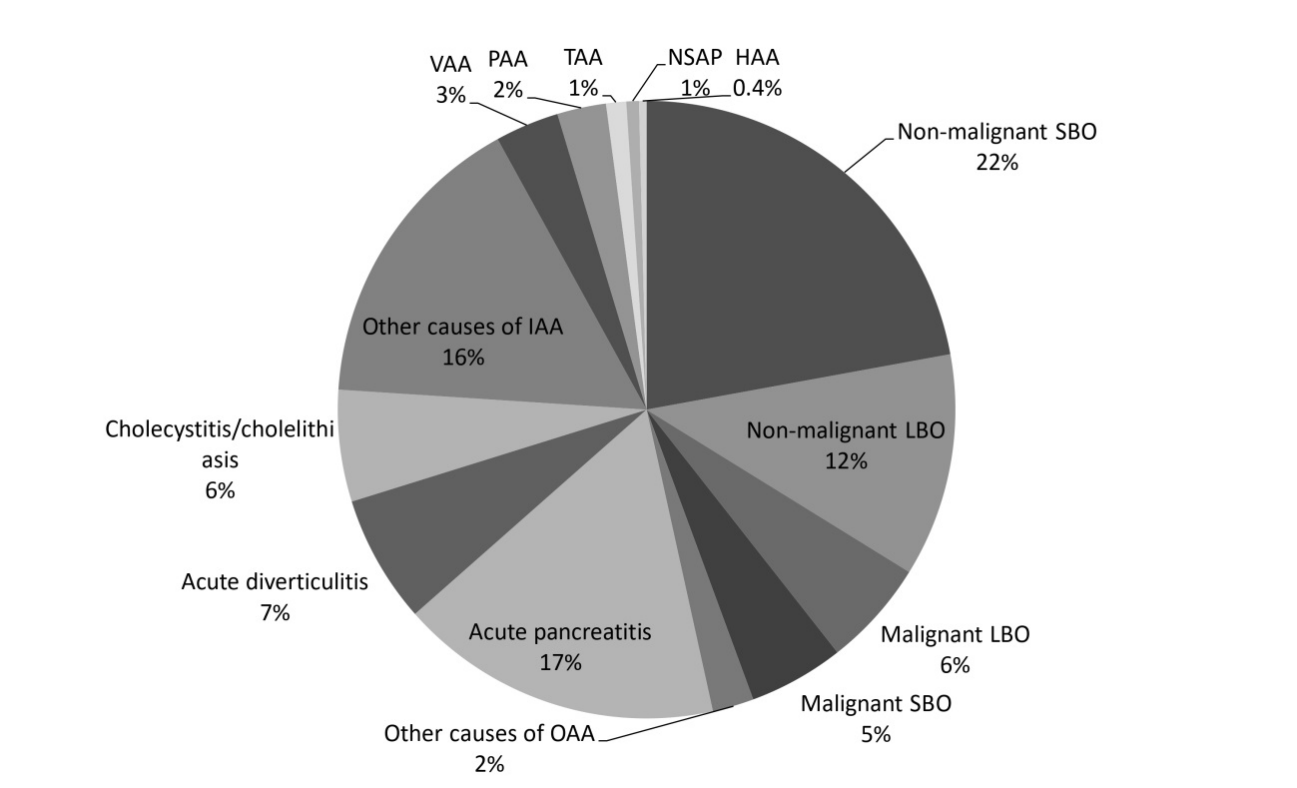
Causes of acute abdomen leading to admission to the intensive care unit.

AA: acute abdomen; LBO: large bowel obstruction; SBO: small bowel obstruction; OAA: obstructive AA; HAA: hemorrhagic AA; NSAP: non-specific abdominal pain; TAA: traumatic acute abdomen; PAA: perforated AA; VAA: vascular AA.

Most of them were admitted with OAA (n=709) and IAA (n=692) (Figure 1). Non-malignant SBO, non-malignant LBO, malignant LBO and malignant SBO were observed, respectively, in 337 (48%), 178 (25%) 85 (12%) and 76 (11%) patients with OAA. The remaining 33 (5%) patients with other causes of OAA had gastric outlet obstruction mainly due to cancer (n=25). 

The main causes of IAA were AP (n=258), acute diverticulitis (n=102) and CC (n=89) (Figure 1). The remaining causes of IAA were mainly due to postoperative intra-abdominal infections, leaks or fistulas (n=126), hepatic abscess (n=19) and acute appendicitis (n=19). Vascular acute abdomen, PAA, TAA and HAA were observed in 51 (4%), 39 (3%), 16 (1%) and 6 (1%) subjects, respectively. 

The two most common causes of VAA, PAA, TAA and HAA were, respectively, mesenteric ischemia (n=25) and splanchnic vein thrombosis (n=20); perforated gastric and duodenal ulcers (n=29) and iatrogenic endoscopic perforations (n=7), blunt abdominal (n=6) and splenic rupture (n=3) and hepatocellular carcinoma rupture (n=2) and retroperitoneal bleeding (n=2).

Overall, 196 (13%) patients died due to septic (n=143), hypovolemic (n=9) and cardiogenic (n=2) shock; advanced cancer (n=38), acute respiratory distress syndrome (n=2), acute myocardial infarction (n=1) and pulmonary embolism (n=1). Five hundred and seventeen (34%) required surgical intervention. The median length of stay (LOS) was 9 [1–101] days (Table 1).

Comparison of demographics, clinical data and outcomes according to the cause of AA is in Table 2. Patients with OAA, VAA and PAA were significantly older when compared to those with other causes of AA. On the contrary, patients with IAA and TAA had lower APACHE II levels at admission when compared to their counterparts with OAA, PAA, HAA and NSAP. In addition, comorbidity, when assessed by CII, or the presence of cancer was higher in patients with OAA, PAA and HAA. In respect to outcomes, surgical intervention, as expected, was required more often in patients with PAA and TAA. Inflammatory AA and TAA had lower mortality rates, whereas LOS was higher in patients with OAA, TAA and PAA (Table 2).

**Table 2:** Clinical features and outcomes of patients admitted to the intensive care unit according to the acute abdomen classification.

	IAA (n=692)	OAA (n=709)	VAA (n=51)	PAA (n=39)	TAA (n=16)	HAA (n=6)	NSAP (n=10)	p-value
Age (years)	62±19	70±17	67±19	66±15	45±20	55±22	55±21	0.0001
Gender (%)								
Male	43%	42%	49%	36%	50%	50%	40%	0.90
Female	57%	58%	51%	64%	50%	50%	60%	
Clinical features								
APACHE II	9.9±5.9	11.6±5.4	10.7±5.7	11.9±6.2	8.5±4.6	12.5±5.1	11.4±7.7	0.0001
CCI	3 [0–10]	5 [0–13]	4 [0–13]	4 [0–10]	2 [0–7]	4 [0–7]	2 [0–6]	0.0001
Cancer (%)	11	35	10	26	13	33	10	0.0001
Outcomes								
Surgery (%)	37	31	22	59	44	33	10	0.0001
LOS (days)	6 [1–101]	12 [1–68]	5 [1–24]	11 [1–68]	12 [2–36]	3 [1–7]	7 [1–24]	0.0001
Mortality (%)	8.8	16	18	23	6.3	17	20	0.0001

IAA: inflammatory acute abdomen; OAA: obstructive acute abdomen; VAA: vascular acute abdomen; PAA: perforated acute abdomen; TAA: traumatic acute abdomen; HAA: hemorrhagic acute abdomen; NSAP: non-specific abdominal pain; APACHE II: acute physiology and chronic health evaluation II; CCI: Charlson Comorbidity Index; LOS: length of stay.

In respect to the most common causes of IAA, acute diverticulitis and CC were seen more frequently in older patients (Table 3). Cholecystitis and/or cholelithiasis were more commonly observed, with higher APACHE II scores and CCI and required more often surgical intervention. Other causes of IAA had more often concurrent cancer. These patients had the longest LOS and higher mortality when compared to other with AP, acute diverticulitis or even CC (Table 3).

**Table 3:** Clinical features and outcomes of patients admitted to the intensive care unit according to the most common causes of inflammatory acute abdomen.

	Acute pancreatitis (n=258)	Acute diverticulitis (n=102)	Cholecystitis Cholelithiasis (n=89)	Other causes (n=243)	p-value
Age (years)	59±19	69±15	71±19	59±18	0.0001
Gender (%)					
Male	46	40	44	40	0.88
Female	54	60	56	60	
Clinical features					
APACHE II score	8.6±4.6	10.2±6.0	11.6±6.2	10.6±6.0	0.0001
CCI	2 [0–10]	3 [0–8]	4 [0–8]	3 [0–11]	0.0001
Cancer (%)	4.3	3.9	5.6	26	0.0001
Outcomes					
Surgery requirement (%)	33*	21	65	37	0.0001
LOS (days)	3 [1–31]	3 [1–32]	4 [1–34]	5 [1–101]	0.0001
Mortality (%)	3.5	6.9	11.2	15.9	0.0001

* Three patients underwent necrosectomy and the remaining cholecystectomy. APACHE II: acute physiology and chronic health evaluation II; CCI: Charlson Comorbidity Index; LOS: length of stay.

Patients with OAA were categorized as malignant and non-malignant LBO and SBO and other causes due to gastric outlet obstruction. Comparison of demographics, clinical variables and outcomes in patients with OAA revealed significant differences, in respect to age, APACHE II score, CCI, frequency of cancer, requirement of surgery, LOS and mortality. In this respect, patients with LBO were older when compared to other with SBO. 

Patients with malignant obstruction had higher APACHE II scores and CCI and longer LOS when compared to those without cancer. When compared to other patients, surgery was more commonly indicated in those with malignant LBO and mortality was significantly lower in non-malignant SBO (Table 4).

**Table 4:** Clinical features and outcomes of patients admitted to the intensive care unit according to the most common causes of obstructive acute abdomen.

	Non-Malignant SBO (n=337)	Non-Malignant LBO (n=178)	Malignant LBO (n=85)	Malignant SBO (n=76)	Other causes (n=33)	p-value
Age (years)	69±17	75±16	72±15	64±16	69±15	0.0001
Gender (%)						
Male	150 (44,5)	68 (38,2)	34 (40)	29 (38,2)	16 (48,5)	-0,542
Female	187 (55,5)	110 (61,8)	51 (60)	47 (61,8)	17 (51,5)	
Clinical features						
APACHE II score	10.8±5.5	12.1±4.8	12.8±5.8	12.5±4.9	11.9±7.0	0.0001
CCI	3.8 [0-12]	4.7 [0-13]	6.4 [1-11]	7.5 [2-12]	5.1 [0-11]	0.0001
Cancer (%)	52 (15,4%)	25 (14%)	-	-	15 (45,5%)	0.0001
Outcomes						
Surgery requirement (%)	112 (33,2%)	38 (21,3%)	42 (49,4%)	17 (22,4%)	10 (30,3%)	0.0001
LOS (days)	11 [1–68]	12 [1–63]	13 [1–55]	13 [1–44]	11 [1–42]	0.0001
Mortality (%)	30 (9%)	27 (15,4%)	21 (25,3%)	26 (34,7%)	9 (27,3%)	

SBO: small bowel obstruction; LBO: large bowel obstruction; APACHE II: acute physiology and chronic health evaluation II; CCI: Charlson Comorbidity Index; LOS: length of stay.

On univariate analysis mortality was associated with age (1.027; 95% confidence interval — 95%CI 1.027–1.037, p<0.0001), APACHE II (1.206; 95%CI 1.170–1.242, p<0.0001), CCI (1.374–1.459, p<0.0001), surgery (1.75; 95%CI 1.291–2.372; p<0.0001) and malignancy (3.3; 95%CI 2.456–4.590; p<0.0001), but only APACHE II (1.173; 95%CI 1.137–1.210; p<0.0001), CCI (1.266; 95%CI 1.182–1.237; p<0.0001) and surgical intervention (1.027; 95%CI 1.027–1.037; p<0.0001) remained significant on multivariate analysis (Table 5). Type of AA was not associated with mortality neither on univariate nor in multivariate analysis.

**Table 5:** Univariate and multivariate analysis of variables associated with mortality in subjects admitted to the intensive care unit with acute abdomen.

Variables	Univariate analysis			Multivariate analysis		
	OR	95%CI	p-value	OR	95%CI	p-value
Age	1.027	1.017–1.037	0.0001			
CCI	1.374	1.294–1.459	0.0001	1.266	1.182–1.237	0.0001
APACHE II	1.206	1.170–1.242	0.0001	1.173	1.137–1.210	0.0001
Malignancy	3.3	2.456–4.590	0.0001			
Surgery requirement	1.75	1.291–2.372	0.0001	1.549	1.093–2.195	0.014

CCI: Charlson Comorbidity Index; APACHE II: acute physiology and chronic health evaluation II.

## DISCUSSION

The demographics, clinical features and outcomes of patients with AA admitted to the ICU, either due to comorbidity, organ failure or disease severity, were retrospectively analyzed. Most of the patients had OAA or IAA with high APACHE II scores and CCI. One third required surgery and 13% of them died during hospital stay. Interestingly, mortality in this cohort was independently associated only with older age, comorbidity and requirement for surgery without any correlation with the type of AA.

Several reports have investigated clinical and laboratory findings as well as outcomes of patients with AA admitted to the ED^4-7,11,14,24,31^ or after emergency surgery^9,25,28-30^. In this regard, the most patients with AAP in the ED were shown to have NSAP or nephrolitiasis^6,11,14^. The majority required no intervention or hospitalization and mortality was negligible^6,11,14^.

Mortality after emergency surgery was shown to vary between 9 to 19.4%^25^ have evaluated outcomes of 748 patients with AA requiring emergency major abdominal surgery. Most of them had adhesions, ischemia or bowel perforation and almost half required ICU admission. Only 9% died in hospital and mortality was associated with severity and comorbidity, respectively assessed by ASA grade and P-POSSUM morbidity. In contrast, Clarke et al.^9^ found higher 30-day mortality rates after emergency surgery, particularly in those subjects older than 80 years, and when compared to our study. Ukkonen et al.^30^ reported similar postoperative 30-day mortality rates, which were also correlated with increasing age, severity and comorbidity including malignancy. 

Few studies have investigated the outcome of patients admitted to the ICU with AA^13,28^. Most of them enrolled patients who developed AA after ICU admission, mostly due to VAA or IAA with a high mortality rate related to late diagnosis and surgical intervention^13,28^. To our knowledge, our study is the first investigation concerning the fate of patients with AA admitted to a dedicated gastrointestinal ICU for either conservative management or in the perioperative period either before or after emergency surgery.

This is a single-centre study with some limitations due to its retrospective design and lack of follow-up after hospital discharge. It is also important to acknowledge that it was performed in a tertiary care center, with a risk of selection bias and overestimation of severity and mortality of those patients who were included in the analysis. Several other studies have dealt with outcomes of subjects hospitalized due to AA with or without requirement of intensive care support^21,23^. Symons et al.^29^ reported outcomes of more than 350 thousand patients who were hospitalized with the diagnosis of life-threatening surgical conditions in different hospitals from the National Health System (NHS) of the United Kingdom from 2000 to 2009. More than half of those patients had OAA and the remaining had miscellaneous causes of IAA, PAA or VAA. Overall, 30-day mortality was 15.8%, varying from 7.44 to 47.5% according to the underlying diagnosis. In accordance with our findings, mortality was higher in those with bowel ischemia, older age and higher Charlson scores. Surgical intervention was also required in roughly 1/3 of the patients. Of note, low mortality rates were observed in institutions with higher availability of ICU and high-dependency beds and better imaging resources. 

Recently, another analysis of the NHS database described an even lower mortality rate, but most of the patients had AP or acute appendicitis with a lower inherent risk of death^23^. In this report, lower mortality was also observed in hospitals with higher levels of medical and nursing staffs, greater number of operating theatres and critical care beds. 

In Brazil, one report from the IT Department of the SUS (DATASUS) evaluating outcomes of patients hospitalized with the codified diagnosis of AA described a crude mortality rate of only 9.62%^21^. Lower rates were even described in another temporal analysis from DATASUS involving only patients with non-traumatic AA^18^. Those discrepant results could be attributed to differences in disease severity and profile and frequency of concurrent comorbidity that were much more frequent in our cohort of critically ill patients.

## CONCLUSION

Finally, the patients admitted to the ICU with AA due to disease severity, organ dysfunction or comorbidity constitute a heterogeneous group of subjects with different prognosis according to the type of AA, age, disease severity, comorbidity and concurrent malignancy. Those latter variables, however, are more correlated to adverse outcomes than the categorized cause of AA leading to hospitalization.
